# Development of a Standardized Communication Intervention Bundle for Use at a Medical Training Hospital Intensive Care Unit

**DOI:** 10.5005/jp-journals-10071-23168

**Published:** 2019-05

**Authors:** Katherine Pollard, Brian Todd Wessman

**Affiliations:** 1 Clinical Emergency Medicine and Clinical Medicine, Indiana University School of Medicine, Indianapolis, IN, USA; 2 Anesthesiology and Emergency Medicine, Washington University in Saint Louis, School of Medicine, St. Louis, MO, USA

## Abstract

**How to cite this article:** Pollard K, Wessman BT. Development of a Standardized Communication Intervention Bundle for Use at a Medical Training Hospital Intensive Care Unit. Indian J Crit Care Med 2019;23(5):234–235.

## SETTING AND PROBLEM

With an aging population and the documented increased usage of critical care medicine (CCM) services, effective and timely communication between the medical team and the patient or surrogate is a paramount milestone for all graduate medical education (GME) trainees. During critical illness, active discussions about patients’ preferences leads to shared decision-making (SDM), diminishes patient and family stress, and facilitates patient centered outcomes.^1^ Medical house staff receive limited formal training with respect to initiating and structuring the quality of life discussions with patients and their families.^2,3^ The GME clinical learning environment review (CLER) program integrates medical education into the tapestry of high quality hospital patient care.

We sought to assess and improve the current state of education regarding patient communication and real-world application in our tertiary academic medical center intensive care unit (ICU). Our multidisciplinary ICU team, consisting of CCM faculty member, fellow, unit clinical specialist, nurse researcher, and bedside nurse, utilized a validated survey to perform a needs assessment. Data from our ICU regarding family experiences and medical team perceptions of patient and family centered care were collected. A literature review of best practices for patient and family communication was also performed. Our needs assessment revealed opportunities for improvement in patient centered communication. Only 63.6% (14/22) of family members reported feeling “very included” in the SDM process, and only 32.5% (25/77) of our staff felt we do “very well” in encouraging patients and families to be involved in SDM conversations. A major identified deficiency was formal documentation of these SDM conversations. In response to our assessment, we developed and implemented an evidence-based, standardized communication intervention bundle (CIB) with a goal to increase the frequency of conducting and documenting family SDM discussions, identifying and documenting surrogate decision makers, improving patient/family satisfaction as well as medical provider professional fulfillment.

## INTERVENTION

**Fig. 1:**
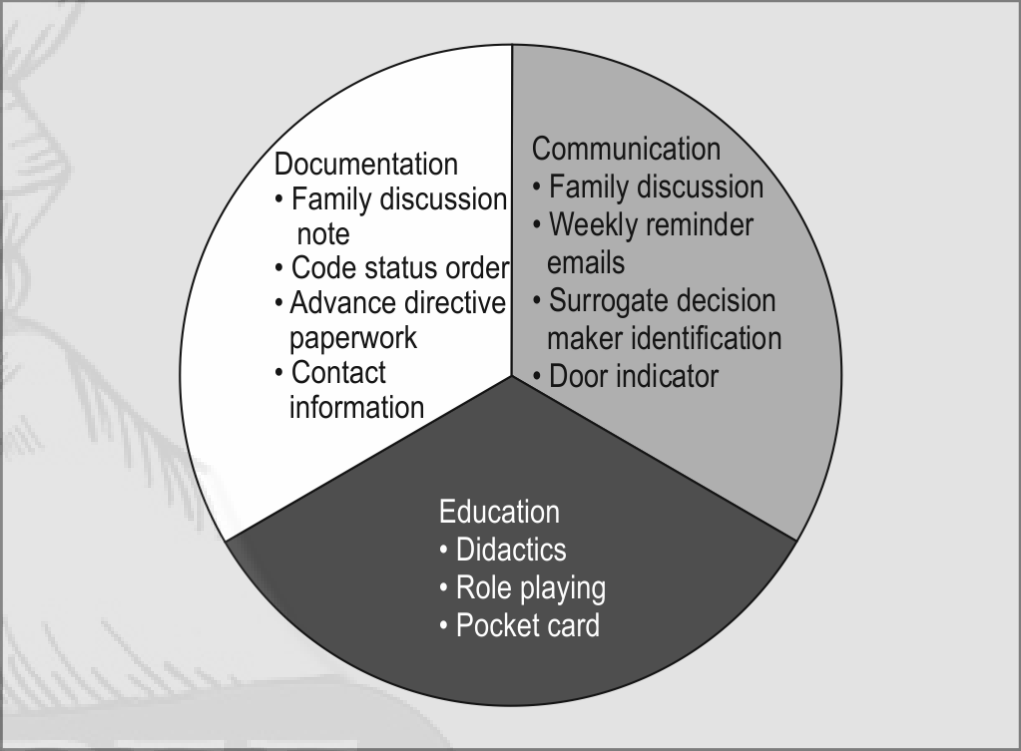
Communication intervention bundle demonstrating the three components documentation, communication, and education

Our CIB focused on 3 elements: education, family discussion, and documentation (Fig. 1).

In keeping with GME CLER goals, the education element consisted of multi-professional didactic sessions and role-playing for CCM faculty, fellows, residents, advance practice providers, and bedside nurses. We created a pocket reference card for longitudinal reference that included model conversation phrases and medical ethics.

The family discussion element consisted of creating an indicator on the patient's glass door signifying discussion completion, follow-up weekly emails from our unit clinical specialist identifying families still in need of a conversation, and actual completion of SDM conversation.

The documentation element consisted of creating a new standardized template for documenting family discussions in our electronic medical record (EMR), updating surrogate decision maker contact information in the EMR, obtaining a copy of advance directive paperwork in the chart, and updating the formal code status order in the EMR.

Patients admitted to our 36-bed ICU with a length of stay >48 hours were screened for inclusion during the 22-week study period. Pre and post intervention data regarding family member experiences were collected using a validated survey tool. Medical team and caregiver perceptions regarding patient and family centered care in the ICU were collected using a validated survey. To audit the implementation of our CIB, weekly chart reviews were performed for the presence of all four documentation elements in the EMR.

## OUTCOME

We successfully completed the multi-professional education component of our CIB across our ICU domain prior to the enrollment phase. Six hundred and eighty patients were screened for eligibility with 394 (58%) meeting the criteria. Total compliance with all four documentation elements was observed in 7% (27/394) of cases. We noted an improvement in SDM discussion documentation in 52% (204/394) of the patients, and an improvement in updated goals of care reflecting the patient's expressed wishes in 32% (66/204) of the patients. The completion of family discussion notes reflected our multi-professional training as advance practice providers (38%; 78/204), critical care fellows (31%; 63/204), and residents (18%; 37/204). By the end of our CIB intervention, bedside nursing was also significantly more likely (65% vs 52%; *p* <0.05) to confirm a formal surrogate decision maker identified by the patient.

Despite its simplicity, implementation of the CIB increased family discussions and identification of surrogates led to appropriate patient centered changes in goals of care. We are still analyzing data regarding patient/family and medical staff satisfaction with this CIB intervention. We will also look at the barriers to CIB implantation and longevity of the intervention.
